# Early detection of hepatocellular carcinoma via no end-repair enzymatic methylation sequencing of cell-free DNA and pre-trained neural network

**DOI:** 10.1186/s13073-023-01238-8

**Published:** 2023-11-08

**Authors:** Zhenzhong Deng, Yongkun Ji, Bing Han, Zhongming Tan, Yuqi Ren, Jinghan Gao, Nan Chen, Cong Ma, Yichi Zhang, Yunhai Yao, Hong Lu, Heqing Huang, Midie Xu, Lei Chen, Leizhen Zheng, Jianchun Gu, Deyi Xiong, Jianxin Zhao, Jinyang Gu, Zutao Chen, Ke Wang

**Affiliations:** 1 Department of Oncology, Xinhua Hospital Affiliated to Shanghai Jiao Tong University School of Medicine, Shanghai, China; 2 BamRock Research Department, Suzhou BamRock Biotechnology Ltd., Suzhou, Jiangsu Province China; 3 Division of Hepatobiliary and Transplantation Surgery, Department of General Surgery, Nanjing Drum Tower Hospital, the Affiliated Hospital of Medical School, Nanjing University, Nanjing, China; 4 Department of Transplantation, Xinhua Hospital Affiliated to Shanghai Jiao Tong University School of Medicine, Shanghai, China; 5 Hepatobiliary Center, the First Affiliated Hospital of Nanjing Medical University, Nanjing, Jiangsu Province China; 6 College of Intelligence and Computing, Tianjin University, Tianjin, China; 7 Department of Software Engineering, Tsinghua University, Beijing, China; 8 National Clinical Research Center for Hematologic Diseases, Jiangsu Institute of Hematology, The First Affiliated Hospital of Soochow University, Suzhou, Jiangsu Province China; 9 Suzhou Known Biotechnology Ltd, Suzhou, Jiangsu Province China; 10 Infectious Disease Department, the First Affiliated Hospital of Soochow University, Suzhou, Jiangsu Province China; 11 Department of Pathology, Fudan University Shanghai Cancer Center, Shanghai, China; 12 Department of Oncology, Shanghai Medical College, Fudan University, Shanghai, China; 13 Department of Pathology, Xinhua Hospital Affiliated to Shanghai Jiao Tong University School of Medicine, Shanghai, China; 14 Department of Interventional Medicine, the affiliated hospital of infectious diseases of Soochow University, Suzhou, 215131 Jiangsu Province China; 15 Liver Transplantation Center, Union Hospital, Tongji Medical College, Huazhong University of Science and Technology, Wuhan, Hubei Province China; 16 Suzhou Key Laboratory of Pathogen Bioscience and Anti-Infective Medicine, Suzhou, Jiangsu Province China

**Keywords:** Hepatocellular carcinoma, Early detection, Cell-free DNA, Circulating tumor DNA, Whole-genome methylation sequencing, Enzymatic conversion, Read level, Neural network, No end-repair enzymatic methyl-seq

## Abstract

**Background:**

Early detection of hepatocellular carcinoma (HCC) is important in order to improve patient prognosis and survival rate. Methylation sequencing combined with neural networks to identify cell-free DNA (cfDNA) carrying aberrant methylation offers an appealing and non-invasive approach for HCC detection. However, some limitations exist in traditional methylation detection technologies and models, which may impede their performance in the read-level detection of HCC.

**Methods:**

We developed a low DNA damage and high-fidelity methylation detection method called No End-repair Enzymatic Methyl-seq (NEEM-seq). We further developed a read-level neural detection model called DeepTrace that can better identify HCC-derived sequencing reads through a pre-trained and fine-tuned neural network. After pre-training on 11 million reads from NEEM-seq, DeepTrace was fine-tuned using 1.2 million HCC-derived reads from tumor tissue DNA after noise reduction, and 2.7 million non-tumor reads from non-tumor cfDNA. We validated the model using data from 130 individuals with cfDNA whole-genome NEEM-seq at around 1.6X depth.

**Results:**

NEEM-seq overcomes the drawbacks of traditional enzymatic methylation sequencing methods by avoiding the introduction of unmethylation errors in cfDNA. DeepTrace outperformed other models in identifying HCC-derived reads and detecting HCC individuals. Based on the whole-genome NEEM-seq data of cfDNA, our model showed high accuracy of 96.2%, sensitivity of 93.6%, and specificity of 98.5% in the validation cohort consisting of 62 HCC patients, 48 liver disease patients, and 20 healthy individuals. In the early stage of HCC (BCLC 0/A and TNM I), the sensitivity of DeepTrace was 89.6 and 89.5% respectively, outperforming Alpha Fetoprotein (AFP) which showed much lower sensitivity in both BCLC 0/A (50.5%) and TNM I (44.7%).

**Conclusions:**

By combining high-fidelity methylation data from NEEM-seq with the DeepTrace model, our method has great potential for HCC early detection with high sensitivity and specificity, making it potentially suitable for clinical applications.

DeepTrace: https://github.com/Bamrock/DeepTrace

**Supplementary Information:**

The online version contains supplementary material available at 10.1186/s13073-023-01238-8.

## Background

Cancer is a leading cause of death, with an estimated 19.3 million new cases and nearly 10 million deaths worldwide in 2020 [1]. Among these, hepatocellular carcinoma (HCC) is one of the most common and deadliest types of liver cancer, accounting for a huge number of cancer deaths globally [1]. HCC is typically associated with chronic liver disease (LD), primarily cirrhosis [2]. Unfortunately, most HCC patients are diagnosed at advanced stages with a median overall survival of only 1–1.5 years [3]. However, early detection of HCC significantly improves the prognosis, with a 5-year survival rate more than 70% [2]. Researchers have been investigating various biomarkers to aid in early detection. One such promising and non-invasive biomarker is cell-free DNA (cfDNA), which is the fragmented DNA in human peripheral blood and other circulating fluids. A small proportion of cfDNA is circulating tumor DNA (ctDNA), which is the DNA derived from tumor cells. Detecting ctDNA in plasma cfDNA has achieved success in diagnosing various cancers including HCC, since ctDNA carries cancer-specific genetic and epigenetic aberrations [4, 5]. Specifically, abnormal DNA methylation changes have been reported in early stage of HCC [6–9].

The methylation sequencing (methyl-seq) technology uses sodium bisulfite to convert the unmethylated cytosines to uracils. While bisulfite treatment is a widely used method for detecting DNA methylation, it requires harsh chemical conditions (in low pH, high temperature, and high concentration salt solution for a long time), leading to DNA damage, fragmentation, and degradation [10]. Particularly, bisulfite treatment has been reported to preferentially damage DNA in hypo-methylated regions with high GC content [11], which was associated with the causes and development of multiple cancers including HCC [12]. Moreover, the bisulfite treatment can exhibit flaws during unmethylated cytosine conversion, potentially reducing their accuracy and sensitivity of cancer detection. To address the limitations of bisulfite treatment, enzymatic conversion-based methyl-seq (EM-seq) has been developed and has shown significant potential in increasing accuracy in detecting cancer with less DNA damage and more CpG sites (CpGs) covered [13]. However, current EM-seq method is inaccurate when applied in cfDNA or degraded DNA (e.g., DNA from formalin-fixed, paraffin-embedded (FFPE) samples), because these DNA fragments contain various lengths of jagged ends [14–16]. Most double-stranded cfDNA carry single-stranded ends, termed a jagged end, and the lengths of jagged ends varied among cfDNA fragments [14]. Since double-strand DNA library construction is used in current EM-seq method, it has to do end-repair proximal to 3’ end of cfDNA using the unmethylated nucleotides before conversion [17]. Therefore, this end-repair process may introduce a considerable amount of unmethylation errors in CpG sites, although the original sites are actually methylated. Moreover, these unmethylated CpGs introduced artificially are difficult to be removed due to the various lengths of jagged ends among cfDNA fragments as mentioned above [14]. To overcome this problem, we are first to develop a new library construction method, called No End-repair Enzymatic Methyl-seq (NEEM-seq).

In recent years, deep learning techniques have been applied to genomic sequence research and cancer detection [18–20]. Most researchers utilize Convolutional Neural Network (CNN) [19, 21], Recurrent Neural Network (RNN), such as Long Short-Term Memory (LSTM) [22] or a hybrid model that integrates the advantages of the CNN and RNN (e.g., CNN + LSTM) [23–25]. However, these models have limited performance that is restricted by the quantity and quality of annotated data. They can only capture the task-specific information contained in supervised labels [26] and hardly learn general deep semantics of genome sequences, which may limit their performance in detecting cancer in read level [27].

To address these issues, we propose DeepTrace [28], a read-level HCC detection model that captures the genetic information of methyl-seq data in a Bidirectional Encoder Representations from Transformers (BERT) like model [29]. In brief, the DeepTrace model was first pre-trained to learn the general semantics of human DNA methylation sequences without any additional annotations. DNA methylation and CpG sites (CpGs) are infrequent throughout the whole genome, but crucial for carcinogenesis. Thus, the methylated CpGs in sequencing reads were recoded, and we modified the Masked Language Model (MLM) task in the pre-training phase, to enable the model to pay more attention to the methylation status of CpGs. The pre-trained DeepTrace was then fine-tuned for the specific task of HCC-derived read identification. A validation cohort containing 130 individuals further demonstrates our proposed DeepTrace model to be powerful in detecting early-stage HCC with low-depth cfDNA NEEM-seq data, providing a potential and affordable solution for clinical applications.

## Methods

### Sample collection

In this study, total 42 pairs of human HCC tissues and para-tumor tissues, 62 HCC plasma samples, 67 LD plasma samples, and 39 healthy plasma samples were collected from three hospitals: the Affiliated Hospital of Infectious Diseases of Soochow University, the First Affiliated Hospital of Nanjing Medical University, and the Xinhua Hospital Affiliated to Shanghai Jiao Tong University School of Medicine. The LD patients include chronic hepatitis (hepatitis B, C, and E), liver cirrhosis, liver damage, and acute hepatitis patients. All HCC and LD patients have undergone a definite clinical diagnosis. None of the HCC patients had received any prior treatment for tumor before blood collection and surgery resection. The healthy volunteers have successfully passed a physical examination indicating the absence of liver disease. Primary tumor and para-tumor tissues were collected during surgical resection. Approximately 10 ml of peripheral blood was collected from each participant at the time of diagnosis by using Cell-Free DNA BCT tube (Streck). The peripheral blood of HCC individuals was collected before surgical resection and treatment. All procedures were approved by ethics committee of three aforementioned hospitals and written informed consent was obtained from all participants. The information about the participants was summarized in Additional file 1.

### DNA isolation and library preparation

Plasma was collected by centrifuging blood at 1600* g* for 10 min at 4 °C and followed by centrifuging at 16,000* g* for 10 min at 4 °C to remove cell debris. Genomic DNA (gDNA) of tissues and cfDNA of plasma were extracted by using blood/tissue DNA magnetic bead extraction kit (GeneOn Biotech). All procedures were conducted according to the manufacturer’s protocol. DNA quantity was assessed using Qubit dsDNA HS Assay (Thermo Fisher Scientific). Extracted cfDNA and gDNA were stored at − 80 °C for ready use.

The gDNA was acoustically sheared to an average size 200–280 bp (peak approximately 250 bp) by using fragmentation device Covaris instrument. Two internal controls, unmethylated lambda and CpGs methylated pUC19, were added in each sample. Forty nanograms gDNA or 5–20 ng cfDNA were subjected to the enzymatic conversion step using Enzymatic Methyl-seq Conversion Module (NEB, E7125S) according to the manufacturer’s protocol. In brief, Tet methylcytosine dioxygenase 2 (TET2) and T4-BGT enzymes were used to protect 5mC and 5hmC from deamination. Subsequently, APOBEC3A converted cytosines, but not the protected forms of cytosines, to uracils. The enzymatic converted DNA was then subjected to Accel-NGS DNA library Kit (Swift Biosciences) for single-strand DNA library preparation according to the manufacture’s protocol. Briefly, tails and truncated adapter 1 were ligated to 3’ end of single-strand DNA. A new DNA strand was generated via the extension step, followed by adding truncated adapter 2 to the 5′ end of DNA. Finally, an indexing PCR step was performed to increase yield of DNA molecules with full-length adapter 1 and adapter 2.

We also used human reference gDNA (NA12878, Coriell) and human cfDNA samples to compare three different methods for methyl sequencing library construction, including NEEM-seq, Enzymatic Methyl-seq (EM-seq), and whole-genome Bisulfite Sequencing (WGBS) which was considered as the gold standard. The input amount of DNA was 40 ng for gDNA and 20 ng for cfDNA, and was consistent across different library construction methods. The cfDNA samples were from the same individual. The EM-seq library was constructed using NEBNext Enzymatic Methyl-seq Kit (NEB, E7120S) according to the manufacturer’s protocol. For WGBS, the DNA samples were converted using EZ DNA Methylation-Lightning Kit (Zymo) according to the manufacturer’s protocol. The bisulfite converted DNA was then subjected to Accel-NGS DNA library Kit (Swift Biosciences) for library preparation according to the manufacturer’s protocol.

### Sequencing, mapping, and DMRs identification

The libraries were paired-end 150 bp sequenced on NovaSeq 6000 sequencers (Illumina). Raw data was filtered using fastp 0.20.1 [30]. Low-quality reads were filtered and adapters were trimmed using default parameters of fastp. In addition, according to the manual of Swift library construction kit, after adapter trimming, 15 bases from the end of read 1 and 15 bases from the beginning of read 2 were trimmed to eliminate the majority of tail sequence. Five bases from the beginning of read 1 and five bases from the end of read 2 were also trimmed. After reads trimming, reads less than 36 bp were removed. Bismark 0.23.0 [31] was used to map the clean reads to the human reference genome (hg38). Reads mapped simultaneously to two or more regions of the genome were removed, and only the unique mapped reads were retained. PCR duplications were identified and removed using Bismark, followed by extraction of methylation status of each site. Deconvolution of cfDNA samples was conducted using MethAtlas [32].

Differential methylation analysis between tumor tissue group and para-tumor tissue group was performed using R package methylKit 1.18 [33]. The 42 pairs of tumor tissues and para-tumor tissues from 42 HCC patients were used. The CpG sites with FDR corrected *p*-value (*q*-value) less than 0.01 and methylation difference greater than 25% were considered as differentially methylated CpG sites (DMCs). Then contiguous DMCs were connected into differentially methylated regions (DMRs). DMRs satisfying all the following conditions were retained: (1) containing at least five DMCs; (2) The distance between DMCs does not exceed 300 bp.

### Data noise reduction

The HCC tumor tissues from resection usually contain other cells besides tumor cells, such as normal hepatocytes, immune cells, and vascular endothelial cells. During model training, if all reads from tumor tissues were labeled as tumor, noise labels will be mixed, which may cause interference and confusion to neural network and affect the training effect. To reduce these noises, reads from HCC tumor tissues were filtered using reads from cfDNA of healthy individuals. The Methylation Continuity Score (MCS) of a given read was defined by the following formula:

$$MCS=\frac{\sum_{i=1}^{L}\left({i}^{2}\times {n}_{i}\right)}{{L}^{2}}$$

where *L* is the number of CpGs within the read; define a block consisting of *i* continuous methylated CpGs in the read, and *n*_*i*_ is the number of corresponding blocks in the read.

The value of MCS ranges from 0 to 1. The higher the MCS value, the higher the methylation level of the read, and the more continuously distributed methylated CpGs, and the less they are separated by the non-methylated CpGs in the read.

For each DMR, the MCS of each read from tumor tissue DNA and from cfDNA of healthy individuals were calculated. Only the reads containing three or more DMCs were used. The maximum and minimum MCS in all reads from cfDNA of healthy individuals were denoted as *S*_max_ and *S*_min_, respectively. If the DMR is hypo-methylated, reads with MCS greater than or equal to *S*_min_ in tumor tissue DNA were removed. If the DMR is hyper-methylated, reads with MCS less than or equal to *S*_max_ in tumor tissue DNA were removed. If the length of a DMR was more than 150 bp, the reads within it were filtered using sliding windows with 150 bp length and 50 bp step size. Perform the above filtering steps for reads in each sliding window.

### Further screening of DMRs

After data noise reduction, the number of retained reads from tumor tissue DNA per DMR per individual varied among DMRs. To screen out DMRs with a high proportion of retained reads in more individuals, we defined an indicator called DMR Universality Score (DUS). The DUS of a given DMR was defined as follows:

$$DUS=\frac{\sum_{i=1}^{n}{t}_{i}}{n}\times d$$

where *n* is the total number of individuals; *t* is the ratio of reads count after filtration to the total read count before filtration in the individual *i*; *d* is the proportion of individuals with *t* > 0 to the total number of individuals. The DUS value ranges from 0 to 1. DUS = 0 indicates that the number of remaining reads after filtration in all individuals is 0. DUS = 1 indicates that no reads were filtered in all individuals. The optimal parameters in the DUS formular were determined by pre-experiments.

DMRs containing more than 200 retained reads (total in all individuals) after filtration were selected. These DMRs were arranged in order of DUS values from large to small, and 10,000 hypo-methylated DMRs (hypo-DMRs) and all hyper-methylated DMRs (hyper-DMRs) were selected for subsequent analysis. Homer [34] was used for annotation of the screened DMRs. The genes that overlapped with DMRs in the upstream and downstream 2-kb region of the transcription start site (TSS) were defined as DMR-related genes. R package “clusterProfiler” [35] was used for GO and KEGG enrichment analysis of DMR-related genes. The *q*-value threshold was set to 0.05.

### Neural Architecture of DeepTrace

The architecture of DeepTrace is similar to BERT model, which is a bidirectional encoder representation model based on Transformer. It consists of an embedding layer, multiple layers of Transformer encoders. The embedding layer learns an embedding matrix to map each token to a fixed-length real-valued vector. These vectors capture the semantic and contextual information of tokens and represent the relationships between tokens in a continuous vector space. Each encoder layer is composed of multi-head self-attention mechanisms and feed-forward neural networks. The self-attention mechanism allows the model to consider all positions in the input sequence simultaneously, effectively capturing the contextual relationships. With the multi-head mechanism, BERT can learn multiple attention representations in parallel, capturing semantic information at different granularities.

In this work, the inputs of BERT are DNA reads that tokenized by n-gram. The hyperparameters of the model are the same as the BERT-base: the number of encoder layers is 12, the number of self-attention heads is 12, the hidden size of embedding is 768, the total number of parameters is 110 M.

### Pre-training of DeepTrace

Eleven million sequences of reads from two cfDNA samples of two LD patients were used in the pre-training phase. Only the reads with mapping quality greater than 30 were used. The paired-end reads overlapping with each other were merged. After merging, only reads containing three or more DMCs were used. To highlight the difference between methylated CpGs and unmethylated CpGs in reads, the “CG” in each methylated CpG site was recoded as “ML”. Each recoded read was served as an input sequence of DeepTrace.

For a sequence (i.e., recoded read), we employed k-mer representation method that has been widely used in biomedical research to tokenize the DNA sequence. In this way, each read was tokenized into multiple consecutive bases that contained rich contextual information. Additionally, we added a special token [CLS] as the first token of sequence like BERT to represent the entire DNA sequence information. In pre-training phase, we only used the MLM task that predicts the masked tokens to learn the contextual representation of DNA methyl-seq sequences. However, the proportion of methylated CpGs in genome was extremely low, which occupies only 1 ~ 2% of the total bases in the whole genome. It is hence difficult for the traditional MLM task that randomly masks 15% of the tokens in each sequence to learn the methylation information. To address this issue, in addition to traditional MLM, we also masked 80% of tokens containing “ML” with methylated CpGs.

During the pre-training phase, we used 11 million sequences of reads from two cfDNA samples of two LD patients. The optimizer we used is adam where learning rate is 1e − 4, β1 is 0.9, β2 is 0.999, and L2 weight decay of 0.01. The dropout probability is 0.1 on all layers. We used a gelu activation in the model. The loss function of pre-training is the mean cross-entropy loss for masked token prediction. We utilized 4 NVIDIA Tesla V100 16 GB GPUs for pre-training with data parallelism. The training process took approximately 30 days to complete.

### Fine-tuning of DeepTrace

After the model pre-training, we conducted the HCC-derived reads (i.e., tumor reads) identification task via model fine-tuning. All of the reads within the DMRs were extracted from bam files. The retained reads from tumor tissues after filtration were labeled as “1,” and reads from cfDNA of LD patients and healthy individuals were labeled as “0.” After noise data reduction, approximate 1.2 million sequences labeled as “1” and 2.7 million clean sequences labeled as “0” were used for fine-tuning. The dataset exhibits a relatively balanced ratio of positive to negative labels at approximately 1:2, no special handling or processing has been applied for data balance. Ninety percent of data was used for fine-tuning training and the remaining 10% of data was used for fine-tuning test. Because the amount of our total training data is large enough, overfitting is not expected to be a problem. During fine-tuning phase, we used the representation of [CLS] token that from last layer of DeepTrace for the final classification. The training objective was cross-entropy loss function. All parameters of the model and the newly added classification layer are involved in the training.

Other public models including LSTM, GRU, CNN + LSTM, and CNN + GRU were also trained to compare with DeepTrace. In order to compare the performance of different models in resistance to noise data interference, original raw data including noise sequencing reads (before data noise reduction) were also used to train models. All the model trainings were executed on Tesla V100 GPUs.

### Model evaluation

Accuracy, f1 score, Matthews correlation coefficient (MCC), receiver operating characteristic (ROC) curve, and precision recall (PR) curve were used to evaluate the performance of models in identifying HCC-derived reads. The total number of reads that used for evaluating model performance was marked as letter “n.” The difference between prediction and real label was described by the number of true positives (TP), true negatives (TN), false positives (FP), and false negatives (FN).

Accuracy calculated the proportion of correct prediction of all reads in both classes. The formula of accuracy was accuracy = (TP + TN)/*n*.

F1 score can be interpreted as a harmonic average of the precision and recall. The precision was the ratio TP/(TP + FP) and the recall was the ratio TP/(TP + FN). The formula for the F1 score was f1 score = 2 * (precision * recall) / (precision + recall). The range of f1 score was [0, 1], where 1 represented the best value and 0 represented worst score. The relative contribution of precision and recall to the f1 score were equal.

MCC was used to measure the quality of binary classification even if the classes were of very different sizes, which can be regarded as a balanced measurement. It took into account true and false positives and negatives. The range of MCC was between − 1 and + 1. A coefficient of + 1 represented a perfect prediction, 0 meant an average random prediction, and − 1 represented an inverse prediction.

ROC curve was used to display the tradeoff between sensitivity and specificity for different thresholds of a model. AUC calculated area under the ROC curve.

PR curve showed the tradeoff between precision and recall for different thresholds of a model. AUPR calculated area under the PR curve.

In the read prediction process, it took about 5 min of running time and about 2 GB of memory on a single Tesla V100 GPU for each cfDNA sample with 1.6X sequencing depth.

### Individual’s cancer risk score

For a given individual, the individual’s cancer risk score (RS) was estimated according to the following formula:

$$RS=\frac{\sum_{p>t}{p}_{i}}{\sum_{i=1}^{n}{p}_{i}}$$

where *p* is the probability predicted by the neural network that a read is derived from ctDNA; *t* is the defined probability threshold of ctDNA, in which a read with *p* more than *t* was considered deriving from ctDNA; *n* is the number of reads. The value of RS ranges from 0 to 1. The higher the RS value, the higher the individual’s cancer risk.

Because the score of cancer risk was affected by the ctDNA probability threshold *t*, searching for proper threshold *t* to estimate cancer risk was essential for HCC detection. The individuals in validation cohort were randomly split into four roughly equal size groups (four folds, Additional file 2: Fig. S1). One of the folds was first chosen to calculate risk score and utilized to search for the best ctDNA probability threshold *t* and the best risk score threshold. The remaining three folds were then combined to serve as a final independent validation cohort to evaluate the performance of HCC detection. This process was repeated four times (Additional file 2: Fig. S1).

### Data mixing simulation

Simulated cfDNA samples were constructed using real sequencing data. Reads were randomly extracted from two sources: (1) cfDNA sequencing data from LD individuals in the independent validation set; and (2) sequencing data of an HCC tumor tissue. This tumor tissue was an independent sample and was not used in the process of DMRs search or model training. The extracted reads from HCC tumor tissue were mixed to cfDNA reads with different proportions. For each ctDNA proportion, the process was repeated 100 times to simulate 100 parallel samples.

### Visualizing attention map of the DeepTrace model

In order to figure out the DNA sequence and methylation patterns that the DeepTrace model focused on, we visualized the attention weight of [CLS] token that from the last layer of the fine-tuned model. Firstly, we applied linear transformations to hidden state of each position in the input sequence to compute query (Q), key (K), and value (V) vectors. Then the attention scores were calculated by using the scaled dot product to measure the similarity between the query vector and the key vector. These attention scores reflect the level of association between the query vector and the key vector. Finally, to ensure that the sum of attention weights is equal to one, the attention scores were typically normalized. This normalization was achieved by applying the softmax function to the attention scores. The formula for attention weights is as follows:

$$Attention\left(Q,K,V\right)=softmax(\frac{Q{K}^{T}}{\sqrt{{d}_{k}}})V$$

where $\sqrt{{d}_{k}}$ is used to maintain the stability of gradient values during the training process. In this paper, we captured the attention weight of [CLS] token that from the last layer of the fine-tuned model, which reflected the importance of each token for read identification. To investigate the distribution of attention weight more intuitively, tumor tissue and non-tumor-derived reads in a certain DMR in the training dataset were used to calculate the average of attention weight in each position of the DMR respectively. For finding which bases are important for HCC-derived read identification, the frequency of each base and the methylation state in a certain DMR in tumor tissues and non-tumor cfDNA samples was calculated and visualized.

## Results

### NEEM-seq overcomes the drawback of EM-seq associated with cfDNA end-repair

**Fig. 1:**
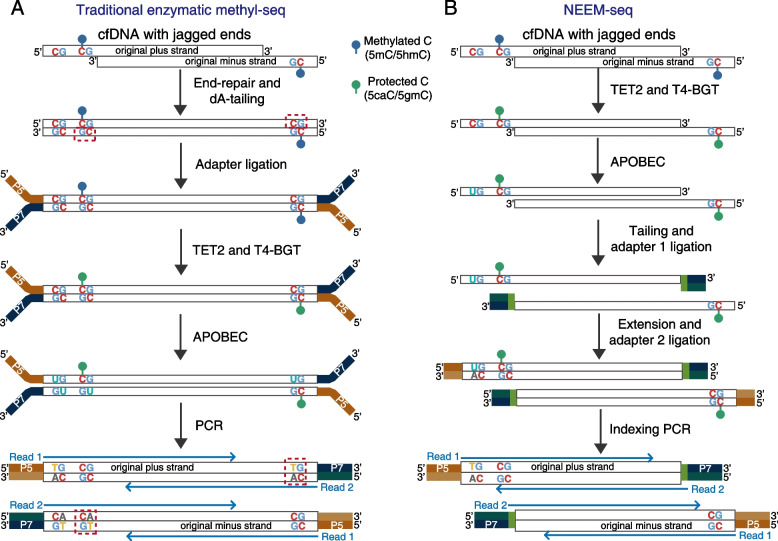
Overview of library construction for traditional enzymatic methyl-seq and NEEM-seq. **A** The process of traditional enzymatic methyl-seq to detect methylated (5mC) and hydroxymethylated (5hmC) cytosines. Firstly, the DNA fragments underwent end-repair and dA-tailing processes, followed by P5 and P7 adapter ligation. Artificially unmethylated cytosines located in CpG sites (indicated by red dashed boxes) were introduced to the cfDNA in end repair. The enzymes TET2 and T4-BGT, oxidized 5mC and 5hmC to 5-carboxycytosine and 5-(β-glucosyloxymethyl) cytosine (5caC/5gmC) to prevent from deamination in the subsequent step. Next, unprotected cytosines were converted to uracils by the deaminase of APOBEC and amplified by PCR for sequencing. **B** The procedures of our NEEM-seq method. The DNA fragments were first converted by TET2 and APOBEC enzymes, followed by the single-strand DNA library construction. Briefly, tails and truncated adapter 1 were ligated to 3′ end of single-strand DNA. A new uracil-free DNA strand was generated through extension, and truncated adapter 2 was added to the other end of DNA. Finally, an indexing PCR step aimed to increase the yield of DNA molecules with full-length adapter 1 (P5) and adapter 2 (P7)

Figure 1A shows that in the traditional enzymatic methyl-seq (EM-seq), the jagged ends of cfDNA were repaired by the unmethylated nucleotides (i.e., A, C, G, and T) before cytosine conversion (Fig. 1A) [17]. In general, the methylation states of the same CpG sites on both strands of the same DNA are supposed to be the same. However, the CpGs in the newly repaired sequence were all unmethylated (red box with dashed line), even though the same CpGs on the complementary strand of the same DNA molecule were methylated. Therefore, this process of end-repair produced artificial unmethylated CpGs to the jagged ends of cfDNA, introducing considerable methylation erasure to the 3’ end. Studies have shown cfDNA and degraded DNA (e.g., DNA from FFPE samples) contained lots of jagged ends [14–16]. Since the front end of reads 2 corresponds to the 3’ end of cfDNA fragments, and the length of jagged ends are various among cfDNA [14], it is expected to be observed that from the tail end to the front end of reads 2, the average methylation ratio will decrease gradually. Conversely, NEEM-seq did not perform end-repair; therefore, the 3' end of jagged DNA was precluded from methylation errors (Fig. 1B). Thus, the average methylation ratio is expected to be evenly distributed on NEEM-seq reads.

**Fig. 2:**
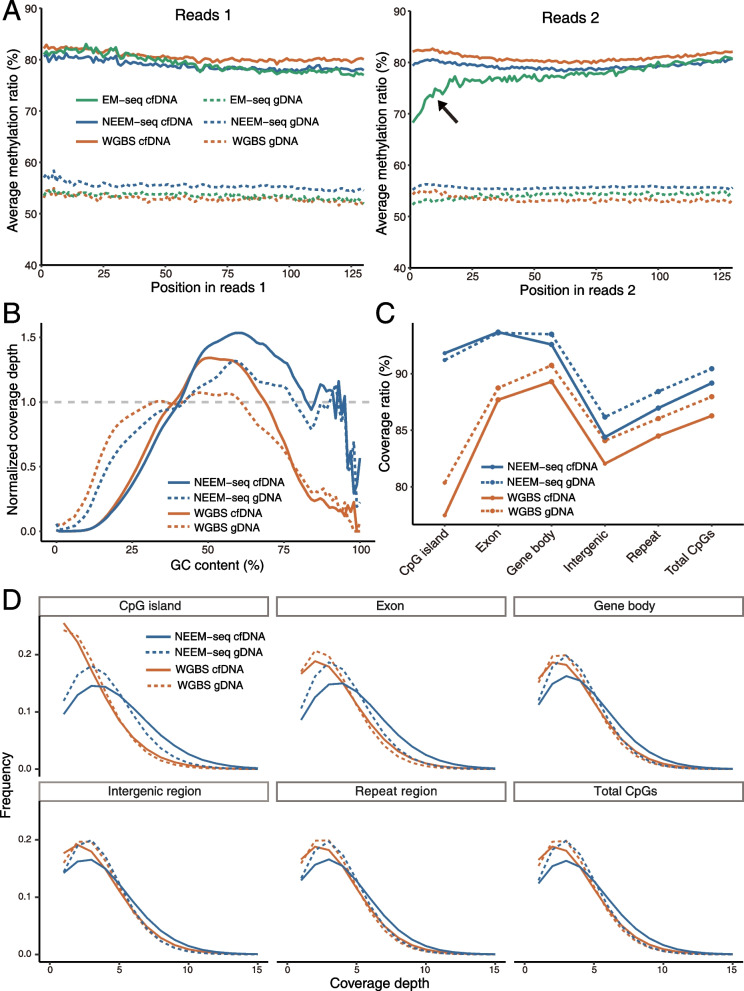
Comparison of results among NEEM-seq, WGBS and EM-seq.** A** Methylation bias plot of reads. This plot shows the average percentage of methylation across each position in the reads 1 and reads 2. The left end of the horizontal coordinate corresponds to the front end of reads. The 15 bases from the front end of reads 2 and tail end of reads 1 have been trimmed before mapping. In the cfDNA EM-seq result (indicated by a black arrow), from the tail end to the front end of reads 2 (i.e., from the 5′ end to the 3′ end of cfDNA fragments), the methylation ratio decreased gradually and obviously. **B** GC bias plot which shows the normalized coverage depth on genomic regions (200 bp sliding window) with various GC contents. **C** Coverage ratio of CpGs in each genomic feature. **D** The frequency distribution of CpGs coverage depth in each genomic feature. The same samples, the same amount of DNA input, and the same sequencing depth were applied across different libraries

To provide the proof of concept, we performed the experiments on the same gDNA and cfDNA samples using three different methyl-seq methods, including EM-seq, NEEM-seq, and WGBS. Methylation ratios were distributed evenly on both reads 1 and reads 2 in all three methods for the human reference gDNA NA12878 (Fig. 2A). This can be attributed to the presence of few and short jagged ends in acoustically sheared gDNA. However, when we sequenced human cfDNA constructed using EM-seq, we observed a gradual and significant decrease in the average methylation ratio (Fig. 2A) from the tail end to the front end of reads 2 (i.e., from the 5’ end to the 3’ end of cfDNA fragments), even after trimming the 15 bases from the front end of reads 2 before mapping. A slight decrease in methylation ratio was also observed from the front end to the tail end of reads 1 (i.e., from the 5′ end to the 3′ end of cfDNA fragments) in the EM-seq results of cfDNA (Fig. 2A), in which the 15 bases from the tail end of reads 1 have also been trimmed. In contrast, methylation ratios of the same cfDNA samples in WGBS and NEEM-seq results were distributed evenly on both reads 1 and reads 2 (Fig. 2A). Notably, both the WGBS and NEEM-Seq used the single-strand DNA library construction method without end-repair. These results suggest that artificial unmethylated CpGs were introduced to the 3′ end of cfDNA fragments during the end-repair process of EM-seq.

With the same DNA input amount and sequencing depth, the coverage ratio and coverage depth of CpGs and the GC bias of NEEM-seq were better than those of WGBS for both gDNA and cfDNA samples (Fig. 2B–D). Additionally, WGBS had much lower coverage on GC-rich regions and CpG islands compared to NEEM-seq (Fig. 2B–D).

### Workflow chart for data generation and analysis by DeepTrace

**Fig. 3:**
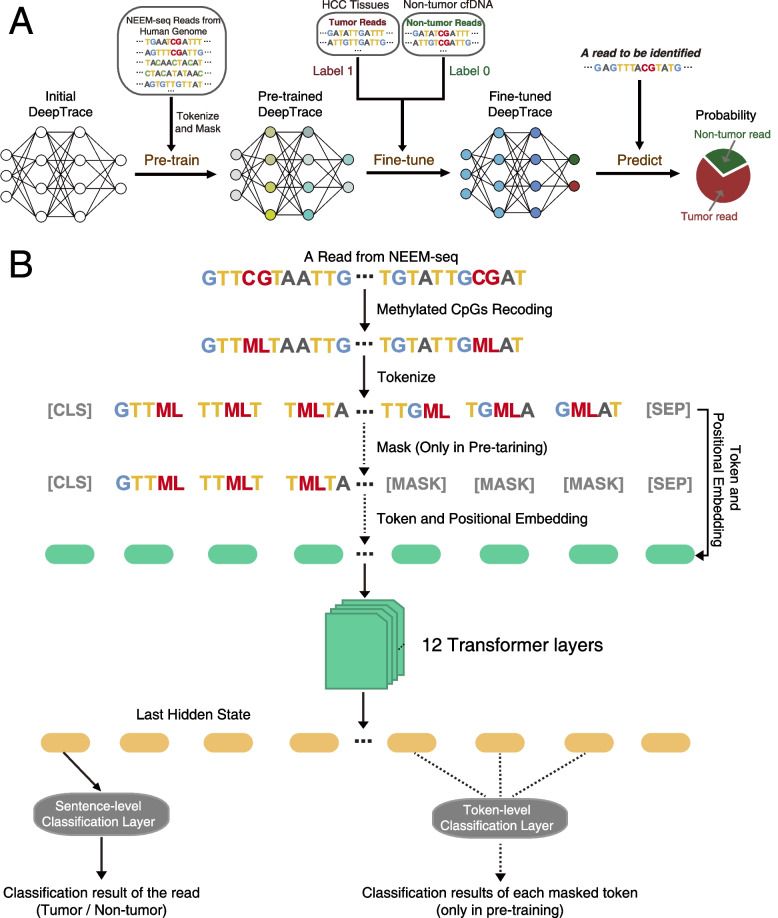
Architecture and characteristics of DeepTrace model.** A** The pre-training, fine-tuning, and prediction process of DeepTrace model using sequencing reads. The NEEM-seq reads from human cfDNA were used to pre-train the DeepTrace model to capture global and transferrable understanding of human genome methyl-seq data. Then the pre-trained model was fine-tuned using tumor reads (i.e., HCC-derived reads) from HCC tumor tissue DNA after noise reduction, and non-tumor reads from non-tumor cfDNA. The fine-tuned model was subsequently used to predict the probability that a read is derived from HCC tumor DNA (i.e., ctDNA). **B** The architecture details of DeepTrace model. The steps indicated by the dashed lines were performed only in pre-training

As shown in Fig. 3A, after reads recoding, tokenize and masking, the whole-genome NEEM-seq reads from human cfDNA were used to pre-train the DeepTrace model to capture global and transferrable understanding of human methyl-seq data. Then the pre-trained model was fine-tuned using tumor reads from HCC tumor tissue DNA after noise reduction (labeled as “1”), and non-tumor reads from non-tumor cfDNA (labeled as “0”). Data noise reduction was conducted on the reads from tumor tissues to retain reads from tumor cells, and remove reads from non-tumor cells in tumor tissues as much as possible (see “Methods” section for details). The fine-tuned model was subsequently used to predict the probability that a read is derived from HCC tumor DNA. The architecture of DeepTrace model is shown in Fig. 3B, and the details are described in the “Methods” section.

**Fig. 4:**
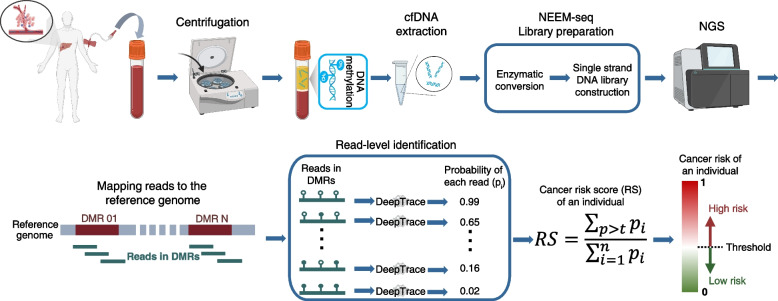
Schematic diagram of the whole process for HCC early detection. The 10 ml peripheral blood was drawn from the individual. After cfDNA extraction, low-depth whole-genome NEEM-seq was performed on the cfDNA sample. DeepTrace model was used to predict the probability of each read within the differentially methylated regions (DMRs). The probability estimated the possibility that a read is derived from ctDNA. The individual’s HCC risk score was calculated by integrating all the reads probability, and the individual’s positive (high risk of HCC) or negative (low risk of HCC) detection result was given

The whole process for HCC early detection is summarized in Fig. 4. A total of 10 ml peripheral blood was drawn from each individual. Low-depth whole-genome NEEM-seq was performed on the cfDNA sample. The DeepTrace model predicted the probability of each read within DMRs. This probability represented the possibility that a read is derived from HCC tumor DNA (i.e., ctDNA). The individual’s HCC risk score was calculated by integrating all the read probability, and the individual was finally classified as either positive (high risk of HCC) or negative (low risk of HCC).

**Fig. 5:**
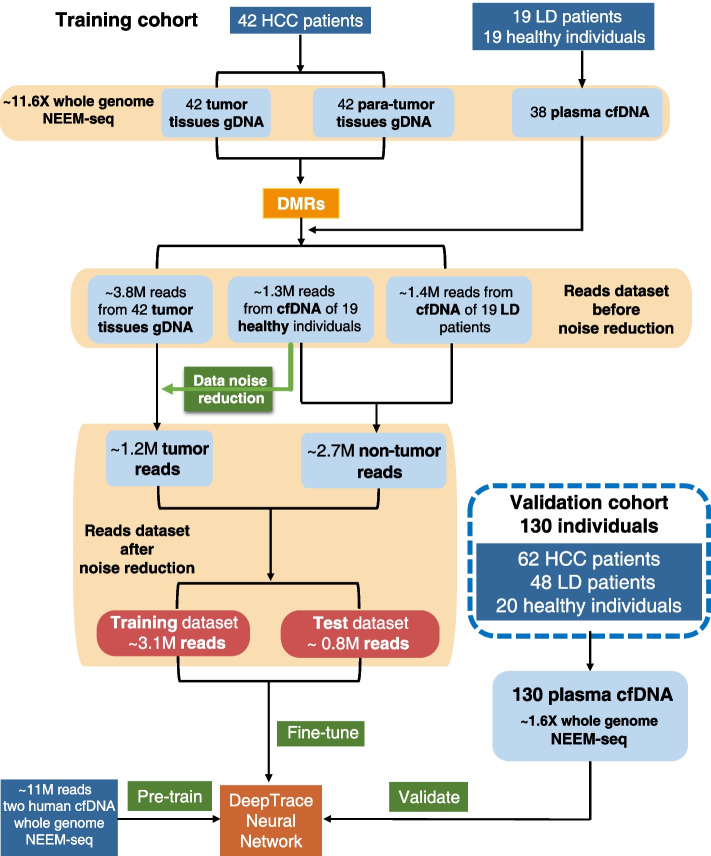
The composition and division of cohort and dataset. Whole-genome NEEM-seq was performed on all samples. About 11 million reads from whole-genome NEEM-seq data were used for DeepTrace model pre-training to capture global and transferrable understanding of human genome methyl-seq data. The pre-trained DeepTrace was then fine-tuned using approximate 1.2 million tumor reads and 2.7 million non-tumor reads after data noise reduction. Before fine-tuning, data noise reduction was conducted on the reads from tumor tissues to retain reads from tumor cells, and remove reads from non-tumor cells in tumor tissues. M: million. LD: liver disease

Figure 5 displays the composition of the cohort and the division of the dataset in detail. Further information is available in Additional file 1. Whole-genome NEEM-seq method was conducted in all samples. The sequencing depth of each of the 84 tissue gDNA samples and 38 cfDNA samples from the training cohort was about 11.6X. Similarly, the sequencing depth of each of the 130 cfDNA samples from the validation cohort was about 1.6X. Notably, each input sample for the DeepTrace neural network was a single read rather than an individual. Therefore, the difference in sequencing depth between the training cohort and the validation cohort did not affect the training and prediction of DeepTrace. A higher depth of the training cohort allowed more read samples for the training of DeepTrace and enabled the identifying of more accurate DMRs. Approximately 11 million reads from NEEM-seq were used for model pre-training to capture global and transferrable understanding of human genome methyl-seq data. The pre-trained DeepTrace was then fine-tuned using approximate 1.2 million tumor reads and 2.7 million non-tumor reads after data noise reduction. Before fine-tuning, the reads from cfDNA of healthy individuals in the training cohort were used to filter the reads from tumor tissue DNA, which removed reads from non-tumor cells in tumor tissues as much as possible (see “data noise reduction” in “Methods” section for details).

**Fig. 6:**
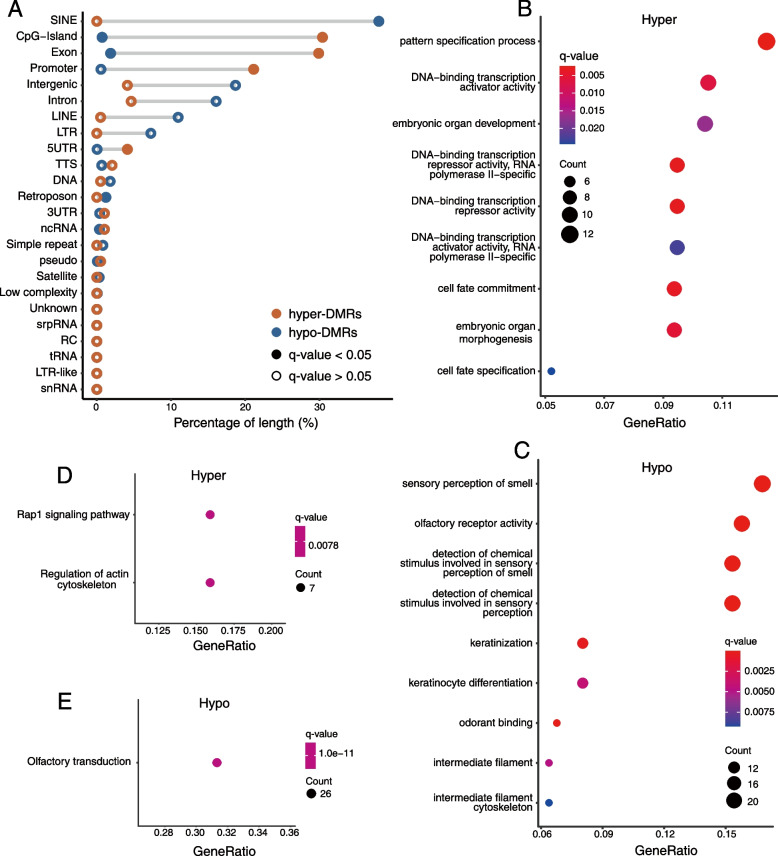
Annotations of DMRs. **A** The genomic features overlap with DMRs. The abscissa axis indicates the proportion of the length of DMRs covering this region to the total length of DMRs. **B ~ C** GO enrichment results of hyper-methylated (**B**) and hypo-methylated (**C**) DMR-related genes. **D ~ E** KEGG enrichment results of hyper-methylated (**D**) and hypo-methylated (**E**) DMR-related genes. The *q*-value denotes the FDR corrected *p*-value

After screening of DMRs (see “Methods” for details), 10,000 hypo-methylated DMRs (hypo-DMRs) and 194 hyper-methylated DMRs (hyper-DMRs) were used in the processes of model fine-tuning and prediction. These hypo-DMRs were significantly enriched in SINE (Alu), CpG island, exon, and retrotransposon, while the hyper-DMRs were significantly enriched in CpG island, exon, promoter, and 5′-UTR (Hypergeometric test, *q*-value < 0.05) (Fig. 6A).

To verify the association of identified DMRs with cancers, GO and KEGG enrichment analysis were performed. The results of GO enrichment (Fig. 6B, Additional file 2: Fig. S2 and Fig. S3) showed that the hyper-DMR-related genes were significantly enriched in several GO terms such as embryonic organ development, cell fate commitment, and DNA-binding transcription activator/repressor (q-value < 0.05). The results of KEGG enrichment (Fig. 6D) showed that these genes were significantly enriched in the Ras-associated protein 1 (Rap1) signaling pathway and the regulation pathway of actin cytoskeleton. Hypo-DMR-related genes were significantly enriched in GO terms such as keratinocyte differentiation, intermediate filament cytoskeleton, and olfactory receptor. They were also significantly enriched in the olfactory transduction pathway (Fig. 6CE, Additional file 2: Fig. S2 and Fig. S3).

The overall genome-wide average methylation ratios of HCC tumor tissues were significantly lower than those of corresponding para-tumor tissues (*p*-value < 0.01). Moreover, 39 out of 42 HCC patients showed that the genome-wide average methylation ratio of a tumor tissue was lower than that of its paired para-tumor tissue (see details in Additional file 1). These findings collectively suggest that global hypo-methylation events have occurred in the genome of most HCC tumor tissues.

Using tissue and cell specific methylation information, deconvolution of cfDNA was performed to figure out the proportion of hepatocyte-derived cfDNA among healthy, LD, and HCC subgroups. The results (Additional file 2: Fig. S4) suggested that the proportion of cfDNA derived from hepatocytes was very similar between healthy individuals and LD patients (mean value was 3% and 4% respectively). However, there was a significant increase in the proportion of hepatocyte-derived cfDNA in HCC patients (mean value 20%; *p*-value < 0.01, Wilcoxon test).

### DeepTrace better identified HCC-derived reads and achieved higher accuracy in detection of HCC individuals than other models and AFP

**Fig. 7:**
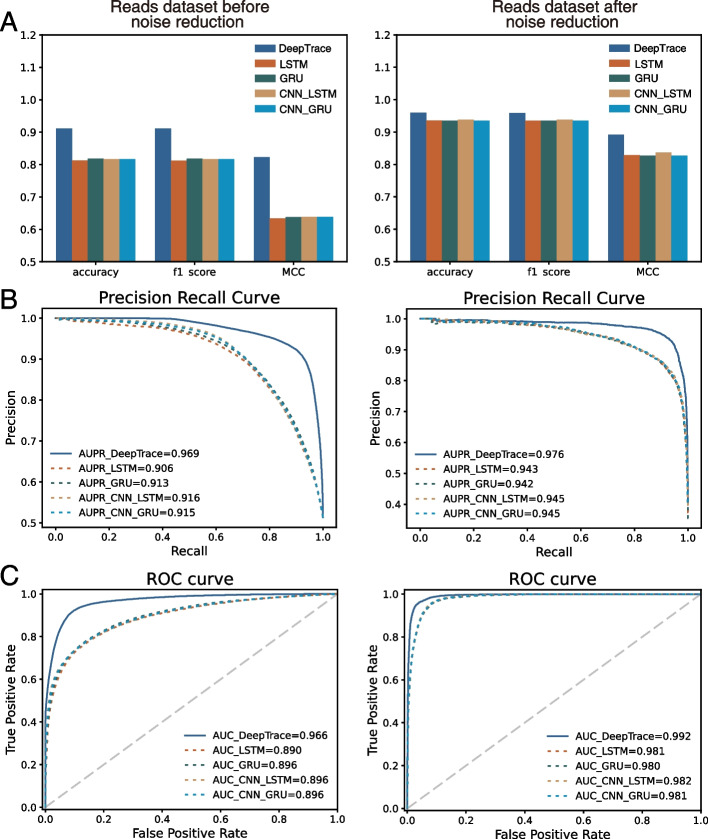
DeepTrace outperformed other models in identifying HCC-derived reads. The performance of DeepTrace in identifying HCC-derived reads was compared with that of LSTM, GRU, CNN + LSTM, and CNN + GRU based on the read datasets before and after noise reduction separately. The read dataset after noise reduction filtered out noise reads according to the rules described in the “Data noise reduction” method. Panel **A** shows the accuracy, f1 score, MCC of different models in identifying HCC-derived reads in the reads test datasets. Panel **B** shows the precision recall curve and panel **C** shows the ROC curve of different models

Accurately identifying ctDNA from cfDNA is essential for early stage of HCC detection. To compare the performance of different models in identifying HCC-derived reads, the read datasets before and after noise reduction were used. The read dataset before noise reduction consisted of all the sequencing reads in the selected DMRs from tumor tissue gDNA and LD and healthy plasmas cfDNA in the training cohort (Fig. 5). The read dataset after noise reduction filtered the noise reads based on the criteria described in the method section “Data noise reduction,” in which around 3.9 million reads were retained. In order to compare different models using the identical data, the same training and test data partitioning was built among different models in the same dataset. The most applied deep learning models in the genomic research (LSTM, GRU, CNN + LSTM, CNN + GRU) were used to compare with our DeepTrace. Parameters such as accuracy, f1 score, MCC, ROC curve, and PR curve were adopted to evaluate the performance of all models. As expected, DeepTrace achieved the best performance in identifying HCC-derived reads in both datasets. As shown in Fig. 7, DeepTrace showed significantly higher accuracy, f1 score, MCC, AUC, and AUPR in the read dataset before noise reduction. Even in the read dataset after noise reduction, DeepTrace still achieved the best performance over all the models, despite the task being easier due to data filtration and reduced noise. The results suggested that DeepTrace could be fine-tuned with high reliability and accuracy to identify HCC-derived reads (e.g., ctDNA) from non-tumor derived reads (e.g., cfDNA). Using DeepTrace model fine-tuned with the read dataset after noise reduction, we identified the reads from the validation cohort and predicted the probability of each read. All predicted values of reads in a single cfDNA sample were then used to estimate the individual’s cancer risk score. The “Methods” section explains the formula used for this purpose.

**Fig. 8:**
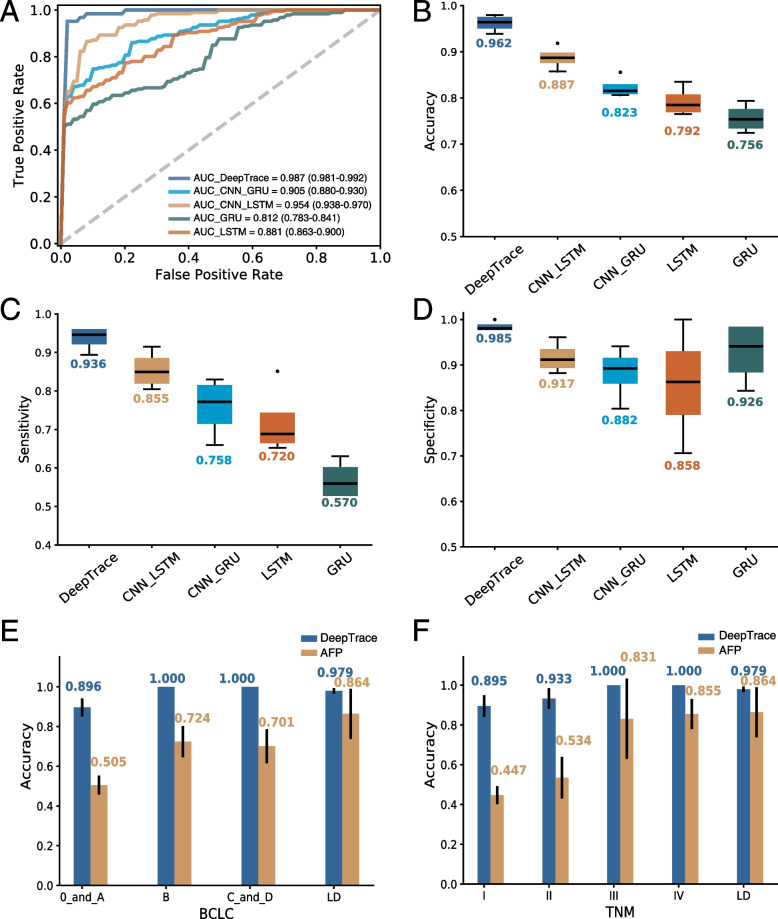
DeepTrace outperformed other models in detection of HCC individuals in the validation cohort.** A** ROC curve of different models on HCC detection (*n* = 130). Blue lines show the average ROC of four-fold cross-validation. **B–D** Accuracy, sensitivity, and specificity of different models on HCC detection (62 HCC, 48 LD, 20 healthy). The boxplots show the data distribution of four-fold cross-validation results. **E,F** Results of DeepTrace and AFP biomarker on HCC detection with BCLC staging system (**E**) and TNM staging system (**F**) (61 HCC, 48 LD). An HCC patient without AFP value was excluded from the analyses in panels **E** and **F**. The error bars indicate the standard deviation of four-fold cross-validation results. The numbers near to the boxplots and histograms in panels **B–F** are the mean values

We also compared the performance of different models in detection of HCC individuals (Fig. 8). The cancer risk score of each individual was utilized for HCC detection in the validation cohort, which included total 130 individuals (62 HCC, 48 LD, and 20 healthy individuals). As shown in Fig. 8AB and Additional file 2: Table S1, DeepTrace showed highest average AUC (98.7% (confidence interval [CI] 98.1–99.2%), accuracy (96.2% (CI 94.5–97.9%)), sensitivity (93.6% (CI 90.7–96.5%)), and specificity (98.5% (CI 97.6–99.5%)) for the detection of HCC individuals in four-fold cross validation datasets.

In order to check the sensitivity of models in different stage of HCC, HCC patients were separated into subgroups based on the Barcelona Clinic Liver Cancer (BCLC) [36, 37] and TNM staging system [38]. Because Alpha Fetoprotein (AFP) is a well-known diagnostic biomarker used in HCC diagnosis, its performance was also assessed in different stages of HCC in the validation cohort. As presented in Fig. 8B–D and Additional file 2: Table S2, DeepTrace showed excellent sensitivities in detection BCLC stage 0 and A (89.6% (85.2–94.0%). It is notable that the only HCC patient with BCLC stage 0 was accurately identified. As expected, the sensitivities for intermediate and advanced stages of HCC detection were higher (stage B: 100.0% (100.0–100.0%); stage C and D: 100% (100.0–100.0%)). Additionally, in the TNM staging system, DeepTrace also showed high sensitivity in early stage (Fig. 8G and Additional file 2: Table S2, TNM stage I: 89.5% (84.2–94.9%)). In comparison, AFP showed much lower sensitivity in early stage of HCC in both BCLC and TNM staging system (BCLC 0 and A: 50.5% (45.9–55.1%); TNM I: 44.7% (40.3–49.2%)). However, AFP still showed a low sensitivity in intermediate BCLC stage B or TNM stage II, at only 72.4% (64.7–80.1%) and 53.4% (43.2–63.7%), which was significantly lower compared to DeepTrace. These results suggested that DeepTrace is more sensitivity in detection of early-stage HCC than AFP.

The specificity of DeepTrace also outperformed that of AFP for LD individuals. In the LD subgroup, the specificity of DeepTrace was significantly higher (97.9%; 95% CI 96.5–99.3%) than that of AFP (86.4%; 95% CI 74.1–98.7%) (Fig. 8EF and Additional file 2: Table S2).

Regardless of HBV infection status or cirrhosis history, DeepTrace consistently demonstrated high accuracy in both HCC and LD subgroup (Additional file 2: Fig. S5AB, Additional file 2: Table S3). The sensitivity of DeepTrace for detecting HCC patients with small (≤ 3 cm) and larger (> 3 cm) tumor size (≤ 3 cm) was 93.8% (95% CI 86.7–100.0%) and 93.6% (95% CI 91.1–96.1%), respectively (Additional file 2: Fig. S5C, Additional file 2: Table S3). In the AFP-negative subgroup (AFP < 20 μg/L), the average accuracy of DeepTrace was 93.4% (95% CI 89.5–97.3%). In AFP-positive subgroup (AFP > 20 μg/L), DeepTrace also demonstrated an accuracy of 96.5% (95% CI 94.1–98.8%) (Additional file 2: Fig. S5D, Additional file 2: Table S4). We conducted a *t*-test to assess the impact of imbalanced gender within our validation cohorts. Our results revealed no significant differences in cancer risk scores between males (*n* = 92) and females (*n* = 38) (*p*-value > 0.05) (Additional file 2: Fig. S5E). DeepTrace model showed excellent performance for both males (mean AUC = 0.994 (CI 99.2–99.6%)) and females (mean AUC = 0.981 (CI 96.8–99.5%)) (Additional file 2: Fig. S5F).

To investigate the minimum read coverage for detecting early HCC using this model, we down sampled the read count for each sample in the validation cohort. The detection results showed that when the remaining specificity (98.5%) is unchanged, the sensitivity (90.3%) of detecting HCC individuals decreased slightly with 3G (1X depth) down-sampled data size. However, the sensitivity (83.8% or lower) decreased rapidly when data size of each sample was down sampled to 1G or lower. Therefore, to achieve a good detection performance, we recommend a minimum coverage depth of 1X for each sample.

In addition to the plasma samples contained in the validation cohort, there were also 13 HCC patients in the training cohort with both tumor tissue and plasma samples available. Among them, 6 were classified as BCLC stage A, 2 as stage B, while the rest as stage C. We also utilized NEEM-seq and the DeepTrace model to test cfDNA samples from these 13 individuals. The results showed that all these 13 individuals tested positive (95% CI of sensitivity: 100 ~ 100%). These results imply that the plasma of these patients contains ctDNA derived from their liver cancer tissues.

To explore the correlation between ctDNA proportion and individual's risk score, reads from an independent tumor tissue were blended with reads from cfDNA to simulate cfDNA samples that contained varying proportions of ctDNA. At a sequencing depth of 1.5X, the result (Additional file 2: Fig. S6A) depicted a significantly positively correlation between the individual’s risk score and the proportion of ctDNA (Pearson correlation coefficient R^2^ = 0.96, *p*-value = 1.5e − 7). This suggests a significant correlation between risk score and the extent of tumor burden. At a sequencing depth of 1.5X, the risk score of simulated cfDNA samples with ctDNA ratio of 2/10,000 was significantly different from that of blank control samples containing no ctDNA (Wilcoxon test, *p*-value < 0.01) (Additional file 2: Fig. S6B). Collectively, these simulation results suggested the estimation of risk score can serve as an effective method for HCC detection at low sequencing depths.

### Attention map of DeepTrace identifies joint patterns of multiple CpG methylation with surrounding DNA sequence

**Fig. 9:**
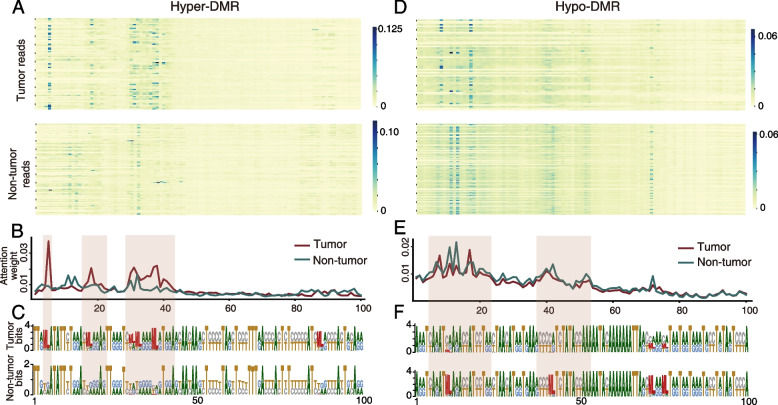
The visualizations of attention weights in hyper- and hypo-DMRs. Panels **A** and **D** show the attention map of a hyper-DMR (chr2: 63,055,387–63055523) overlapping OTX1 and a hypo-DMR (“chr2: 104,265,134–104265334”) overlapping an Alu element. Darker colors represent higher attention weights. Panels **B** and **E** display the average weight of attention in each position of reads in hyper- and hypo-DMR. Red line denotes the reads from tumor tissues and green line indicates the reads from non-tumor cfDNA. Panels **C** and **F** show the frequency of each base in all reads from tumor tissues and non-tumor cfDNA. The methylated CpG sites were marked with letter “ML.” Larger letter represents higher frequency. Regions with high attention weights were marked with red rectangles

To overcome the common “black-box” problem of deep learning models and investigate how DeepTrace distinguishes between HCC-derived and normal reads, we tried to interpret the deep learning model of DeepTrace by investigating its neural network details. The attention map of the DeepTrace was generated to visualize the important regions (blue color) that contribute to the model decision in a hyper-DMR overlapping Orthodenticle Homeobox 1 (OTX1) gene and a hypo-DMR overlapping an Alu element respectively (Fig. 9AD). Our results suggested DeepTrace only focused on specific small regions containing DMRs. Average attention weight of each base position (Fig. 9BE) showed these regions were mainly located in the position 4–6, 16–22, 29-43nt of hyper-DMR and in the position 5–23, 38-53nt of hypo-DMR. All these regions contained DMCs. In the hypo-DMR, the CpGs in the non-HCC-derived reads were almost fully methylated (marked with letter ML), while in HCC-derived reads, the CpGs were almost unmethylated (Fig. 9C, regions marked with red rectangles). Conversely, in the hyper-DMR (Fig. 9F, regions marked with red rectangles), CpGs of the HCC-derived reads were most fully methylated, but not in non-HCC-derived reads. These results suggested that DeepTrace was able to find important regions in DMRs based on its attention mechanism, and DeepTrace successfully distinguished HCC-derived reads by combining multiple CpG methylation information with surrounding DNA sequence together.

## Discussion

DNA methylation sequencing has achieved significant attentions due to its potential to enhance cancer detection sensitivity through genomic methylation profiling [39–41]. In this study, we developed an enzyme-based methylation sequencing technology without end-repair called NEEM-Seq, to achieve single-base resolution and high-fidelity epigenetic profiling of DNA. Furthermore, we developed DeepTrace, a deep learning-based approach to detect early stage of HCC by identifying HCC-derived reads from plasma cfDNA sequencing reads.

### The disadvantage of WGBS, EM-seq, and the advantage of NEEM-Seq

Sodium bisulfite-based methylation sequencing methods, such as WGBS, are widely used to detect methylated and unmethylated cytosines in DNA [42–44]. However, harsh condition of sodium bisulfite treatment has been reported to damage DNA, and preferentially damage on genomic regions with high GC content and low methylation level [45, 46]. Compared with the methyl-seq method based on enzymatic conversion, WGBS exhibits a lower coverage depth on GC-rich regions and CpG islands [17, 47, 48]. Our results (Fig. 2B–D) are consistent with these findings. However, the methylation status of GC-rich regions, such as CpG islands, is critical for the regulation of oncogene and tumor suppressor gene, and carcinogenesis [12, 49]. Thus, the damage and uneven coverage of DNA introduced by bisulfite conversion may reduce the accuracy of cancer detection based on cfDNA methyl-seq.

Although traditional enzymatic methyl-seq methods detect methylation information of cytosines in a mild condition and avoid the problem of DNA damage [17], they introduce unmethylation errors in CpGs for DNA containing jagged ends (e.g., cfDNA and degraded DNA [14–16]) due to the end-repair before cytosine conversion [17]. Because the lengths of jagged ends vary among cfDNA fragments [14], these artificially introduced unmethylated CpGs are challenging to remove according to sequencing results. Thus, these unmethylation errors in CpGs could distort the methylation pattern of reads and the methylation ratio of CpGs. This distortion then may influence the DMR identification, model training, and read identification, decreasing the accuracy and precise of cancer detection. In comparison, NEEM-seq removes the end-repair step and avoids the errors in CpGs of the jagged ends of cfDNA. Our results showed that methylation ratios of cfDNA in NEEM-seq results were evenly distributed on reads, suggesting that NEEM-seq can provide more accurate methylation profiling for cfDNA-based cancer detection than traditional enzymatic methyl-seq methods with end-repair.

Studies have shown an elevation of jagged cfDNA in the plasma of HCC patients compared with non-tumor controls, and in the urinary of bladder cancers or controls, the proportion of jagged cfDNA was much higher than in the plasma [14, 50]. Therefore, the unmethylation errors in these liquid biopsies would be more serious if the traditional enzymatic methyl-seq methods with end-repair were utilized. Although the content of jagged cfDNA in other cancers is needed to be tested, NEEM-seq is expected to be a more accurate method for cfDNA methylation sequencing in the cancer detection based on liquid biopsy.

NEEM-seq only induces base changes at unmethylated cytosines, thus it was expected to maintain most of the cfDNA intact. Therefore, we can extract additional information including cfDNA fragmentation pattern and copy number variations aside from cfDNA methylome. This suggests NEEM-seq has the potential for blood-based multi-omics applications via a single library construction with low cost. Multi-omics data has been demonstrated to be able to improve the specificity and sensitivity of complex diseases such as cancers [51–53]. However, multi-omics data usually requires different sequencing strategies. Recently, Chen et al. developed an HCC detection model based on multi-omics data by constructing methylation sequencing library and whole-genome sequencing library separately [51], which made the experiments and sequencing complex, time-consuming and expensive. Therefore, integrating multi-omics data from our NEEM-seq is expected to be an easier method with lower cost and higher effectiveness for cancer detection.

### Some DMR-related genes have been found to be closely related to cancers including HCC

Over 30% of hypo-DMRs are located in the Short Interspersed Elements (SINEs), which in almost all of them consist of Alu elements. For instance, a DMR (chr2: 104,265,134–104265334) overlapping an Alu element was found to be demethylated in tumor tissues (Fig. 9). SINEs belong to the retrotransposon, whereas Alu elements belong to the most abundant class of SINEs. They are primate specific and constitute 11% of the human genome [54]. Alu elements have often been used as surrogate markers of global DNA hypo-methylation [55]. The demethylation of Alu elements occurs in aging and cancer processes and has been linked with gene reactivation and genomic instability [56]. Alu elements can activate oncogenic pathways in HCC [57], and studies have identified Alu hypo-methylation as increased risk factors for cancers [58].

The hypo-DMR-related genes were significantly enriched in the olfactory transduction pathway, which mainly included olfactory receptor (OR) genes. Furthermore, OR activity was also one of the significantly enriched GO terms. ORs are not exclusively expressed in the olfactory sensory neurons, but also observed in all other human tissues [59]. ORs have been shown to be involved in the modulation of cell–cell recognition, migration, proliferation, the apoptotic cycle, and other processes [59]. Additionally, ORs are highly expressed in various cancer tissues compared with normal tissues, making them potential diagnostic and therapeutic targets [59].

The genes related to hyper-DMRs were significantly enriched in the “DNA-binding transcription activator/repressor” GO term mainly belong to the transcription factor (TF) gene family. Cancer requires constitutive expression of TFs for growth and survival, and many TFs are critical for carcinogenesis [60]. For instance, a DMR (chr2: 63,055,387—63,055,523) overlapping Orthodenticle Homeobox 1 (OTX1) gene was hyper-methylated in tumor tissues (Fig. 9). This gene encodes a member of the bicoid sub-family of homeodomain containing transcription factors, which contributes to HCC progression by regulating the ERK/MAPK pathway [61]. Another example is that the promoter of spalt-like transcription factor 3 (SALL3) gene was hyper-methylated in HCC tumor tissues in this study. The SALL transcription factors are composed of a zinc finger motif and participating in embryonic development [62]. The SALL family also contributes to cellular apoptosis, angiogenesis, invasion, and metastasis of tumors [62]. The downregulation of the expression of SALL3 gene has been reported in HCC [63].

The Rap1 signaling pathway was one of the enriched pathways for hyper-DMR-related genes. Targeting Rap1 signaling and its regulators could potentially control carcinogenesis, metastasis, chemoresistance, and immune evasion [64]. The Rap1 signaling pathway has been found to be closely associated with the HCC tumor-infiltrating immune and clinical prognosis [65].

### The outperformance of DeepTrace in identifying HCC-derived reads and detecting early stage of HCC

CNN or RNN (such as GRU and LSTM) based models have been widely applied in various biological fields, including genomic sequencing [19, 20, 23–25, 66, 67]. However, CNN struggles to obtain long-range contextual information from DNA sequence, and both CNN and RNN rely on a large amount of annotated data, limiting their performance by the quality of that data. In addition, these models can only capture the task-specific information found in supervised labels [26]. Thus, these models are difficult to learn general deep semantics of genome sequences [27], which may limit their performance in read-level HCC detection. Consequently, instead of using CNN or RNN-based models, we proposed a DeepTrace model based on BERT. BERT has overcome the above limitations [29] and based on recent studies, has reached state-of-art performance in identifying cis-regulatory elements [27]. Our study demonstrated that DeepTrace achieved superior performance in identifying HCC-derived reads compared to the most common models available (LSTM, GRU, CNN + LSTM, and CNN + GRU). In order to make the model suitable for methyl-seq data and HCC detection, DeepTrace was first pre-trained to learn the general semantics of human DNA methylation language via self-supervised training, and the pre-trained DeepTrace model was then fine-tuned to specific task of HCC-derived read identification. To focus the DeepTrace model better on the methylation status of CpGs, we recoded the methylated CpGs present in the reads. Additionally, we provided a modification to the MLM task during the pre-training phase.

The traditional cancer detection methods, which depend on NGS and methylation, rely on the methylation levels calculated by averaging across all cfDNA molecules at each site or region. A block of cfDNA molecules is utilized to calculate the β methylation ratio. However, due to the extremely low proportion of ctDNA, the detection of small discrepancies in the average measurement values becomes challenging even with high sequencing depth (typically more than 2000X), leading to a low signal-to-noise ratio [39]. DeepTrace read-level identification method integrates the methylation information from multiple CpGs, and the sequence information surrounding the CpGs in a single read, to identify each cfDNA molecule individually and independently. The neural network predicts the probability that a read is derived from ctDNA, and it is not affected by other reads. This enables detection of a low proportion of ctDNA even at a low sequencing depth. At the sequencing depth of 1.5X, the read mixture simulation experiments suggest that DeepTrace can detect ctDNA with proportion as low as 2/10,000. The high-depth targeted methyl-seq on the DMRs based on multiplex PCR or probe hybridization capture is expected to further improve the detection performance of DeepTrace.

By accurately identifying rare signal of HCC-derived reads and calculating the individual’s cancer risk score, DeepTrace achieved high sensitivity at 93.6% and high specificity at 98.5% in distinguishing HCC and non-HCC individuals at 1.6X sequencing depths, which suggested it was a low-cost but effective cancer detection method. In LD individuals, DeepTrace still exhibited a high specificity of 97.9%. Moreover, in the early stage of HCC, DeepTrace achieved substantial sensitivity (BCLC stage 0 and A, 89.6%; TNM stage I, 89.5%). This result reinforces the idea of employing DeepTrace as a novel strategy for HCC early detection. In clinical practice, AFP biomarker is often utilized for cancer diagnosis, although its reported sensitivity in cancer detection is quite low [51]. Likewise, in our study, the sensitivity of AFP in early stage of HCC was much lower than DeepTrace. Previous studies have shown that the sensitivity of HCC detection when combing AFP with ultrasonography is generally only 48–75% [68]. All these findings suggested DeepTrace is a much more precision method than traditional AFP biomarker and ultrasonography. Nonetheless, more samples and further clinical trials are required to evaluate the method further.

The simulation experiments results suggest that the individual’s risk score has a significant positive correlation with the proportion of ctDNA. As a result, the DeepTrace method has the potential in various applications such as minimal residual disease (MRD) detection, ctDNA dynamic monitoring during treatment, and relapse risk monitoring. Further clinical trials are needed to validate the feasibility and performance of our approach in these various applications.

### DeepTrace identified HCC-derived reads by focusing on multiple CpGs methylation and their surrounding DNA sequence

The outperformance of DeepTrace was partially attributed to its utilization of novel BERT networks and BERT’s attention mechanism. The attention map demonstrated that DeepTrace paid attention to regions where methylation states of CpGs differed between tumor tissues and non-tumor samples. This suggests that DeepTrace comprehends DNA methylation language and recognizes sequence features related to HCC through self-pre-training and fine-tuning training. As a result, this ensures the accurate identification of HCC-derived reads from cfDNA reads without relying solely on one single CpG site’s information. Instead, DeepTrace combined methylation states in multiple CpGs to identify HCC-derived reads. The use of multiple CpG sites could potentially reduce the probability of false judgments caused by technical noise errors (PCR, enzymatic conversion, or sequencing). For instance, we assume that the false ratio of methylation in a single CpG site due to technical noise error is 1%, while the chance of two CpG sites being false simultaneously is only 0.01%. Furthermore, DeepTrace also considered the DNA sequence surrounding CpGs to determine the HCC-derived reads. This further reduces the false ratio of ctDNA judgment, suggesting that DeepTrace is effective in resisting technical noise error and reinforcing the model’s robustness.

### DeepTrace could be easily applied to other cancers and multi-cancer detection

Previous studies have demonstrated the significant potential of cfDNA methylation profiling to detect cancers [8, 41, 69, 70]. Here, we validated the promising potential of cfDNA methylation profiling using our DeepTrace technology for HCC detection. Furthermore, we posit that DeepTrace can potentially be applied to detect other types of cancer using cfDNA from liquid biopsies, such as plasma, urine, and cerebrospinal fluid. However, this requires validation. Notably, a recent large multi-cancer detection program has reported 54.9% sensitivity and 99.3% specificity across various stages and different kinds of cancer types using a methylation-based approach [71], highlighting the promising potential of cfDNA methylation signatures for multi-cancer detection. Therefore, we predict that DeepTrace can be customized to multi-cancer classification based on the source tumor-of-origin or tissue-of-origin of each cfDNA molecule, although this would require the identification of tumor-specific or tissue-specific markers. Owing to the extensive influence of biological factors and medication on methylation status, it is crucial to design and execute carefully controlled studies to evaluate the clinical applicability of DeepTrace when searching for tumor or tissue-specific markers.

## Conclusions

In this study, we propose NEEM-seq as a novel methylation library construction method. The NEEM-seq method overcomes the drawback of traditional EM-seq caused by end-repair and is expected to generate a more precise methylation profile of cfDNA. In addition, we develop a deep-learning model for early detection of HCC based on read-level ctDNA identification by using plasma cfDNA, and the model has exhibited an outperformance with low coverage depth of NEEM-seq data. Future prospective studies with larger sample size are needed to confirm the clinical utility of our model.

### Supplementary Information

**Additional file 1.** Samples_information.

**Additional file 2:** **Fig. S1.** Schematic diagram of cross-validation evaluation in validation cohort. **Fig. S2.** GO enrichment networks of hyper- and hypo- DMRs. **Fig. S3.** GO enrichment trees of hyper- and hypo- DMRs. **Fig. S4. **Cell type decomposition of cfDNA samples with 11.6X and 1.6X sequencing depth. **Fig. S5.** DeepTrace accuracy of HCC detection in different subgroups with different HBV status, cirrhosis history, tumor sizes, AFP concentration and gender. **Fig. S6.** The risk scores of simulated mixed samples constructed using real sequencing data. **Table S1.** Performance of different models in HCC individual detection in the validation cohort (n=130). **Table S2.** DeepTrace and AFP performance in different stages of HCC and LD patients in the validation cohort (*n*=109, 61 HCC, 48 LD). **Table S3.** DeepTrace performance in HCC individual detection with different HBV status, cirrhosis history and tumor size in the validation cohort (*n*=110, 62 HCC, 48 LD). **Table S4.** DeepTrace and AFP performance in different subgroup of AFP concentrations in the validation cohort (*n*=109, 61 HCC, 48 LD).

## Data Availability

The raw sequence data reported in this paper have been deposited in the Genome Sequence Archive in National Genomics Data Center, China National Center for Bioinformation / Beijing Institute of Genomics, Chinese Academy of Sciences (GSA-Human: HRA004780) that are publicly accessible at https://ngdc.cncb.ac.cn/gsa-human [72]. The source code of DeepTrace has been uploaded and is available from GitHub (https://github.com/Bamrock/DeepTrace) [28]. The other data generated or analyzed during this study are included in this published article and its additional files.

## Abbreviations

PLC: Primary liver cancer
HCC: Hepatocellular carcinoma
LD: Liver disease
HBV: Hepatitis B virus
FFPE: Formalin-fixed, paraffin-embedded
NGS: Next-generation sequencing
cfDNA: Cell-free DNA
ctDNA: Circulating tumor DNA
BCLC: Barcelona Clinic Liver Cancer
CNN: Convolutional Neural Network
RNN: Recurrent Neural Network
LSTM: Long Short-Term Memory
GRU: Gated recurrent units
BERT: Bidirectional Encoder Representations from Transformers
DMRs: Differentially methylated regions
DMCs: Differentially methylated CpG sites
MCS: Methylation Continuity Score
DUS: DMR Universality Score
TSS: Transcription start site
MCC: Matthews correlation coefficient
CI: Confidence interval
AUC: Area under the curve
ROC: Receiver operating characteristic
PR: Precision recall
TP: True positives
TN: True negatives
FP: False positives
FN: False negatives
RS: Risk score
WGBS: Whole-Genome Bisulfite Sequencing
EM-seq: Enzymatic methyl-seq
AFP: Alpha Fetoprotein
MLM: Masked Language Model
