# Supplementary material for: Early detection of hepatocellular carcinoma via no end-repair enzymatic methylation sequencing of cell-free DNA and pre-trained neural network
Source: Genome Med. 2023 Nov 8;15:93. doi: 10.1186/s13073-023-01238-8 (PMC10631027; doi:10.1186/s13073-023-01238-8)
Supplement: Supplementary file 2 — Additional file 2: Fig. S1. Schematic diagram of cross-validation evaluation in validation cohort. Fig. S2. GO enrichment networks of hyper- and hypo- DMRs. Fig. S3. GO enrichment trees of hyper- and hypo- DMRs. Fig. S4. Cell type decomposition of cfDNA samples with 11.6X and 1.6X sequencing depth. Fig. S5. DeepTrace accuracy of HCC detection in different subgroups with different HBV status, cirrhosis history, tumor sizes, AFP concentration and gender. Fig. S6. The risk scores of simulated mixed samples constructed using real sequencing data. Table S1. Performance of different models in HCC individual detection in the validation cohort (n=130). Table S2. DeepTrace and AFP performance in different stages of HCC and LD patients in the validation cohort (n=109, 61 HCC, 48 LD). Table S3. DeepTrace performance in HCC individual detection with different HBV status, cirrhosis history and tumor size in the validation cohort (n=110, 62 HCC, 48 LD). Table S4. DeepTrace and AFP performance in different subgroup of AFP concentrations in the validation cohort (n=109, 61 HCC, 48 LD). [file 13073_2023_1238_MOESM2_ESM.pdf]

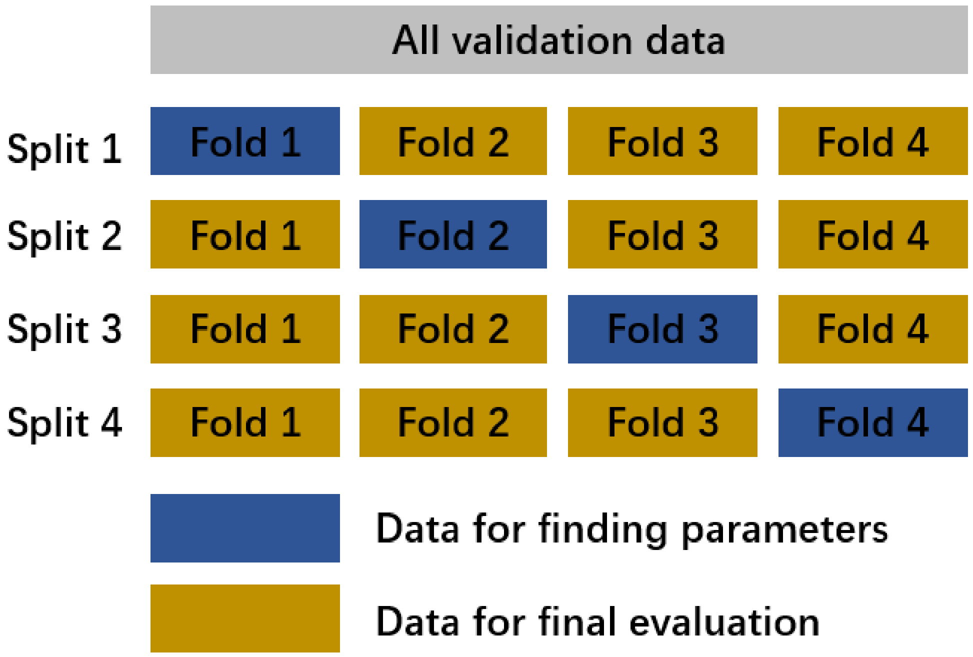

Fig. S1. Schematic diagram of cross-validation evaluation in validation cohort. The individuals in validation cohort were randomly split into four roughly equal size groups (four folds). One of the folds was first chosen to search for the best ctDNA probability threshold t and the best risk score threshold. The remaining three folds were then combined to serve as a final independent validation cohort to evaluate the performance of HCC detection.

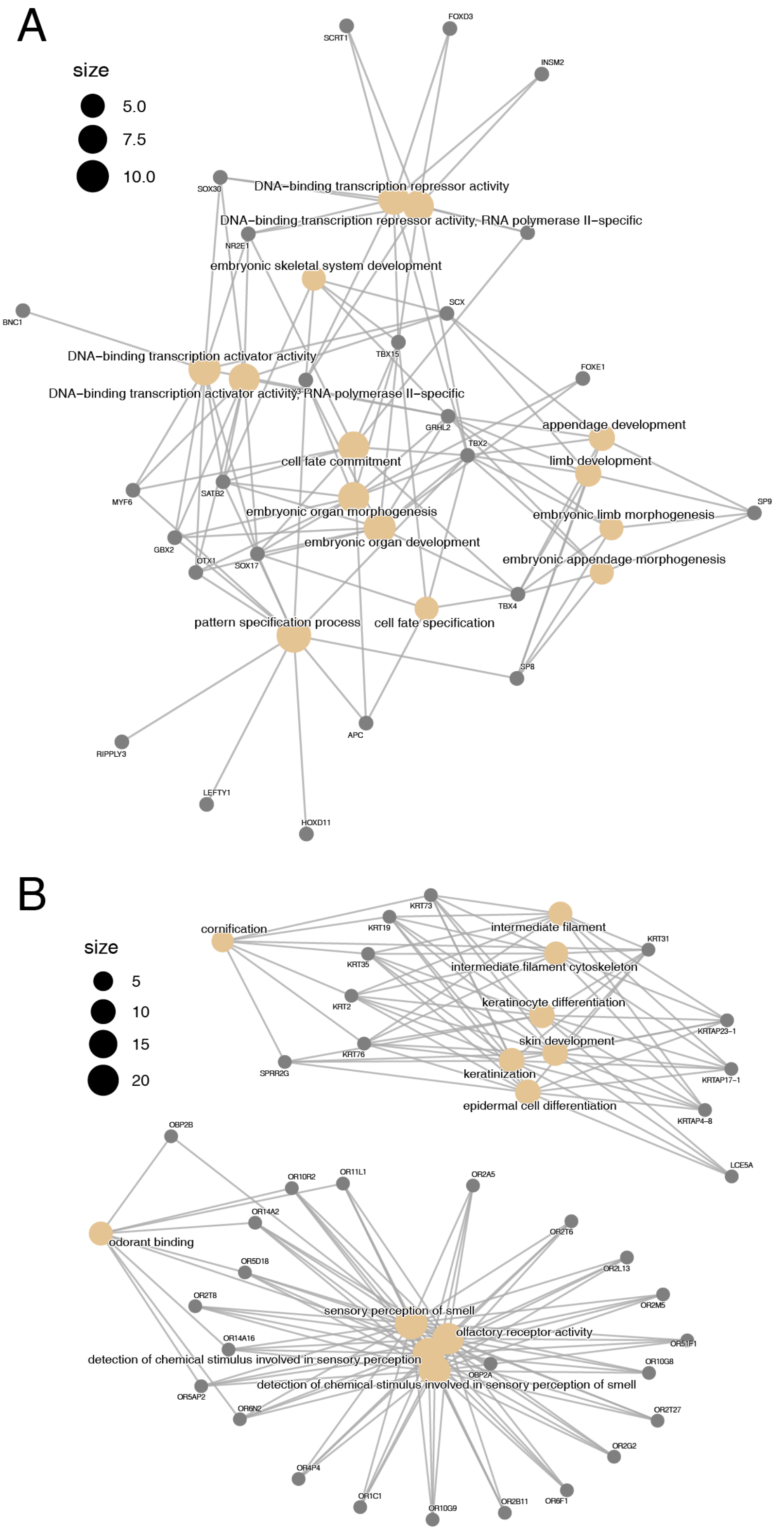

Fig. S2. GO enrichment networks of hyper- (A) and hypo- (B) DMRs.

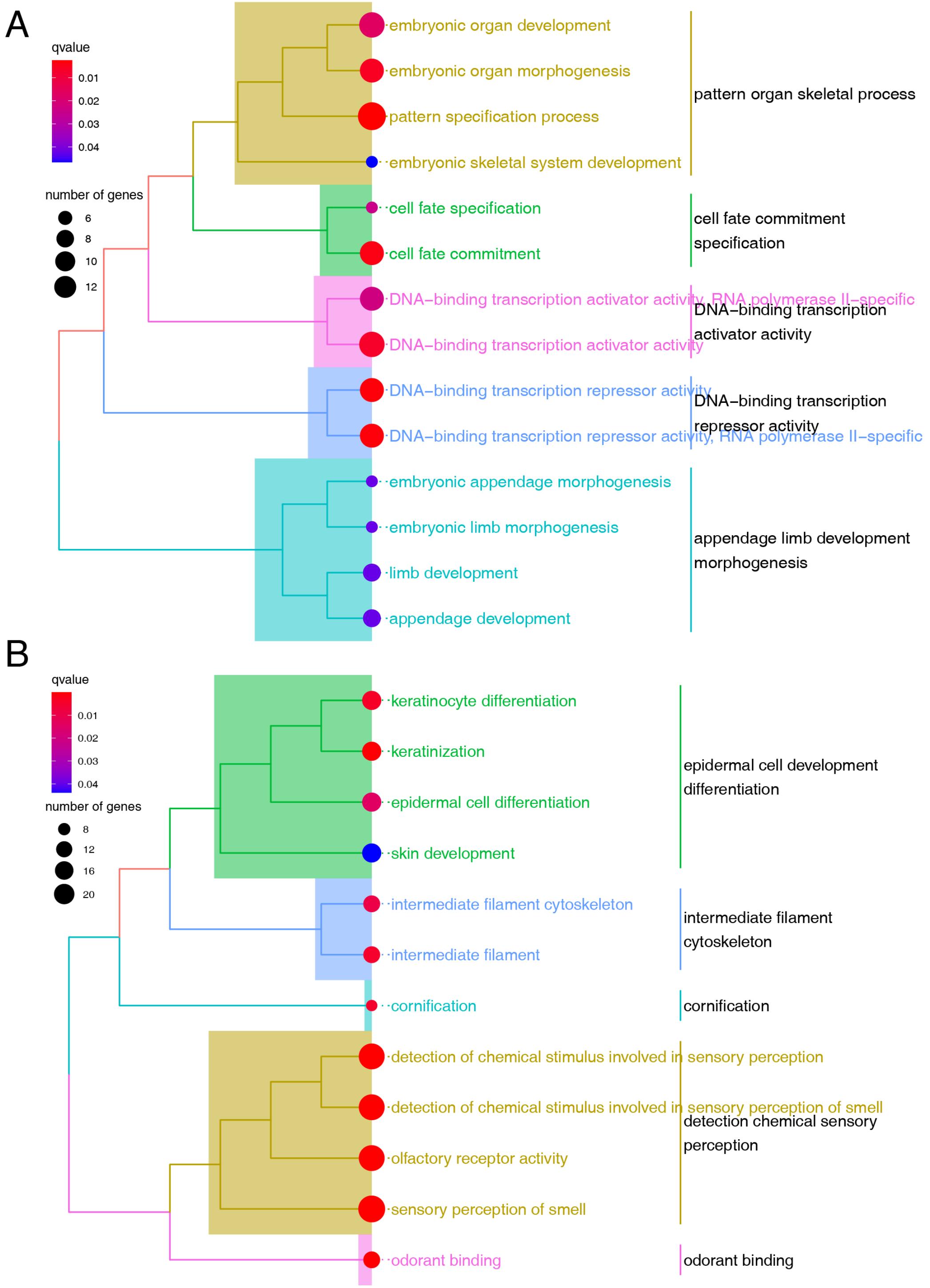

Fig. S3. GO enrichment trees of hyper- (A) and hypo- (B) DMRs.

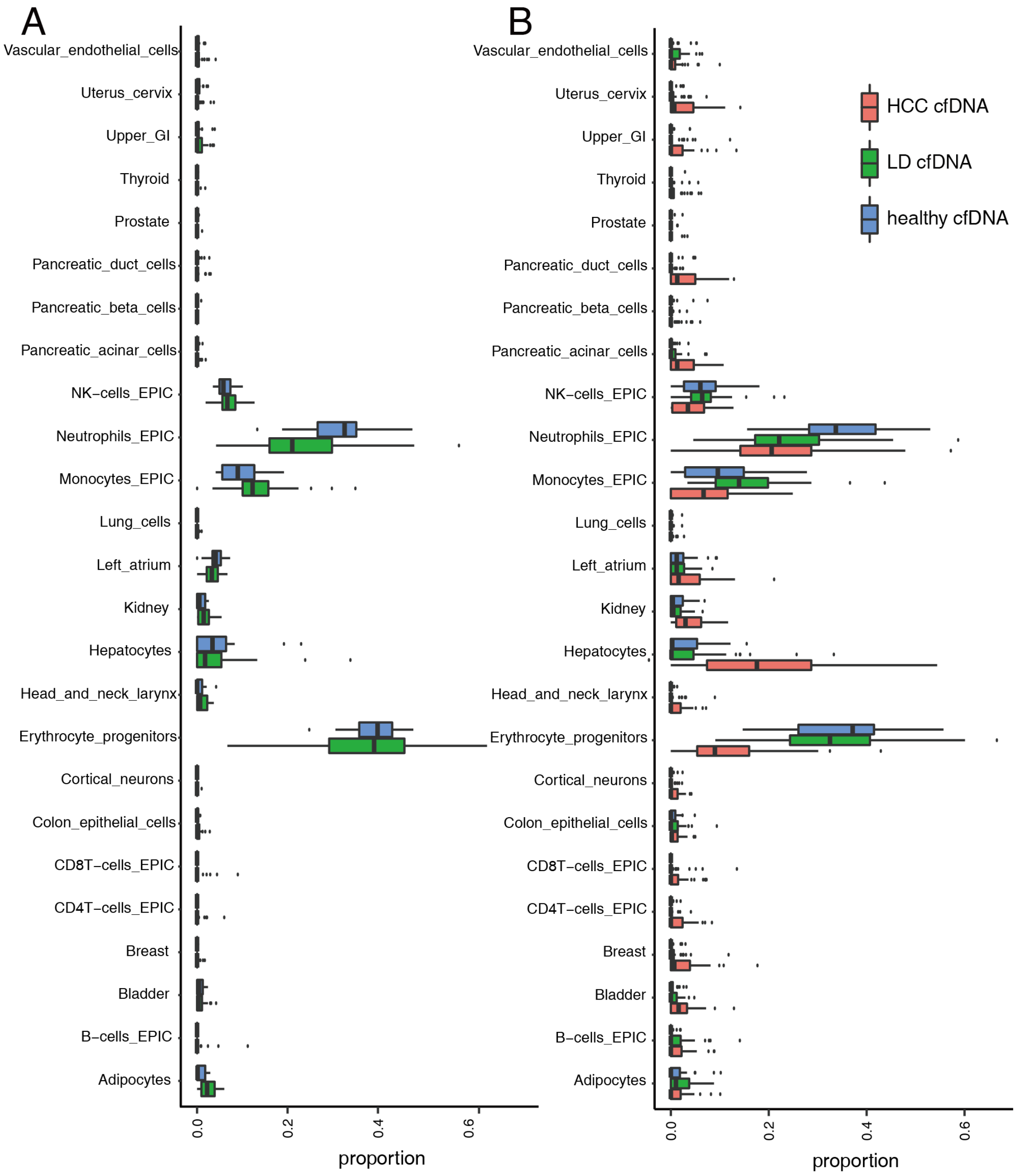

Fig. S4. Cell type decomposition of cfDNA samples with 11.6X (A) and 1.6X (B) sequencing depth.

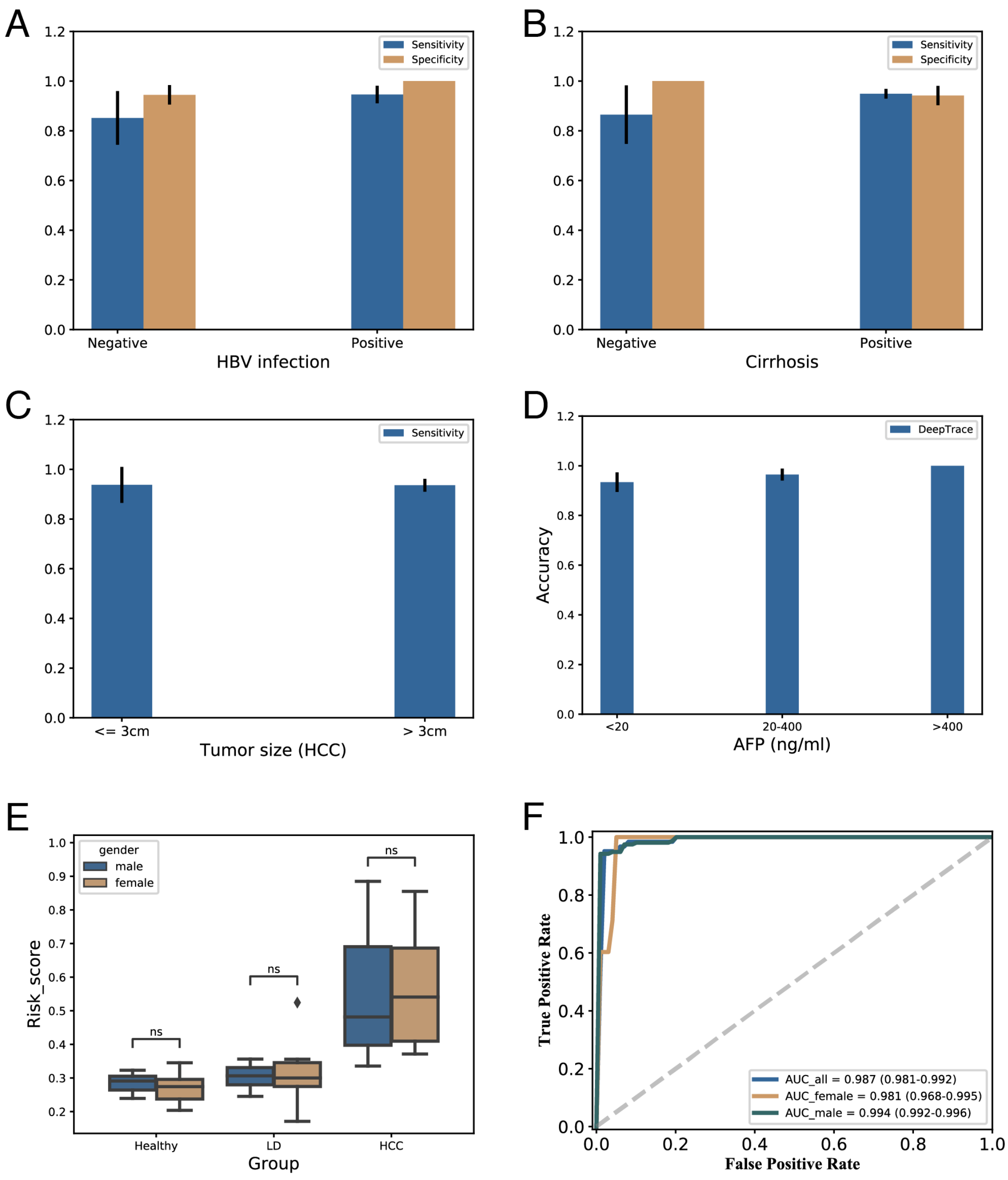

Fig. S5. DeepTrace accuracy of HCC detection in different subgroups with different HBV status (A), cirrhosis history (B), tumor sizes (C), AFP concentration (D) and gender (EF).

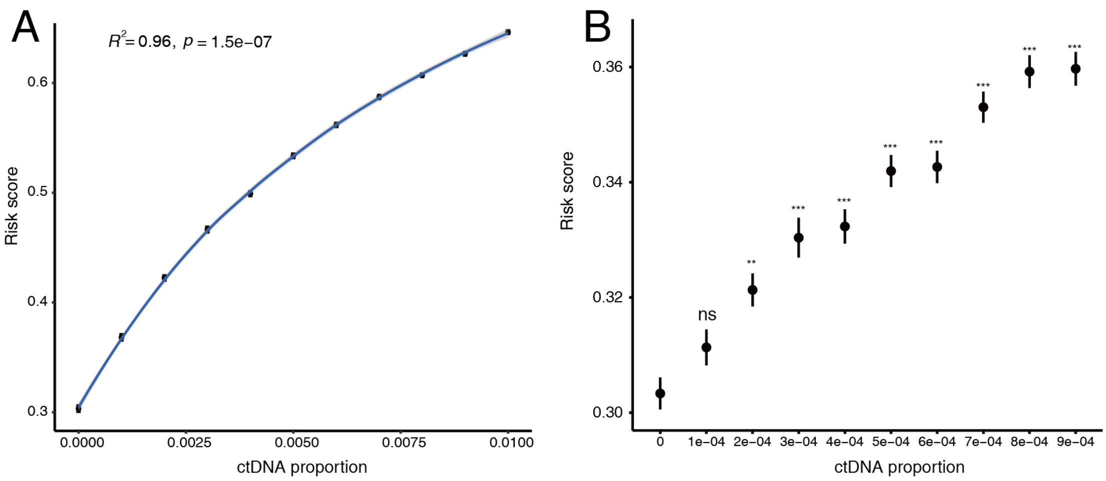

Fig. S6. The risk scores of simulated mixed samples constructed using real sequencing data. (A) The relationship between risk score and ctDNA proportion. The bars depict mean ± standard error. The blue line is the fitting curve. (B) Comparison of risk scores of simulated samples with blank controls. The simulated samples were at 1.5X sequencing depth and contained various proportions of ctDNA. The bars depict mean ± standard error. **: $p < 0.01$; ***: $p < 1e-3$.

Table S1: Performance of different models in HCC individual detection in the validation cohort (n=130). The numbers outside the parentheses are the mean value, and the numbers inside the parentheses are the 95% confidence intervals.

	AUC	Accuracy	Sensitivity	Specificity
DeepTrace	98.7% (98.1%-99.2%)	96.2% (94.5%-97.9%)	93.6% (90.7%-96.5%)	98.5% (97.6%-99.5%)
CNN_LSTM	95.4% (93.8%-97.0%)	88.7% (86.3%-91.2%)	85.5% (80.7%-90.2%)	91.7% (88.4%-94.9%)
CNN_GRU	90.5% (88.0%-93.0%)	82.3% (80.1%-84.5%)	75.8% (68.4%-83.3%)	88.2% (82.6%-93.9%)
LSTM	88.1% (86.3%-90.0%)	79.2% (76.2%-82.3%)	72.0% (63.2%-80.8%)	85.8% (73.6%-98.0%)
GRU	81.2% (78.3%-84.1%)	75.6% (72.7%-78.6%)	57.0% (52.4%-61.7%)	92.6% (86.1%-99.2%)

Table S2: DeepTrace and AFP performance in different stages of HCC and LD patients in the validation cohort (n=109, 61 HCC, 48 LD). The numbers outside the parentheses are the mean value, and the numbers inside the parentheses are the 95% confidence intervals.

Note: An HCC patient without AFP value was excluded from this analysis.

**Stage System**	**Subgroup**	**Number**	**DeepTrace**	**AFP**
**BCLC Stage**	0 and A	37	89.6% (85.2%-94.0%)	50.5% (45.9%-55.1%)
	B	11	100.0% (100.0%-100.0%)	72.4% (64.7%-80.1%)
	C and D	13	100.0% (100.0%-100.0%)	70.1% (61.7%-78.5%)
**TNM Stage**	I	22	89.5% (84.2%-94.9%)	44.7% (40.3%-49.2%)
	II	23	93.3% (88.2%-98.4%)	53.4% (43.2%-63.7%)
	III	6	100.0% (100.0%-100.0%)	83.1% (63.4%-100.0%)
	IV	10	100.0% (100.0%-100.0%)	85.5% (78.1%-92.9%)

**non-HCC**	Liver disease (LD)	48	97.9% (96.5%-99.3%)	86.4% (74.1%-98.7%)

Table S3: DeepTrace performance in HCC individual detection with different HBV status, cirrhosis history and tumor size in the validation cohort (n=110, 62 HCC, 48 LD). The numbers outside the parentheses are the mean value, and the numbers inside the parentheses are the 95% confidence intervals. LD: liver disease.

	Group	Numbers	Sensitivity/Specificity
HBV negative	HCC	7	85.2% (74.6%-95.8%)
	LD	19	94.5% (90.6%-98.3%)
HBV positive	HCC	55	94.6% (91.1%-98.0%)
	LD	29	100.0% (100.0%-100.0%)
Cirrhosis negative	HCC	10	86.5% (75.0%-98.0%)
	LD	31	100.0% (100.0%-100.0%)
Cirrhosis positive	HCC	52	94.9% (93.0%-96.8%)
	LD	17	94.2% (90.4%-98.0%)
Tumor size (HCC)	<= 3cm	10	93.8% (86.7%-100.0%)
	> 3cm	52	93.6% (91.1%-96.1%)

Table S4: DeepTrace and AFP performance in different subgroup of AFP concentrations in the validation cohort (n=109, 61 HCC, 48 LD). The numbers outside the parentheses are the mean value, and the numbers inside the parentheses are the 95% confidence intervals.

Note: An HCC patient without AFP value was excluded from this analysis

AFP concentration (μg/L)	Group	Numbers	Accuracy of DeepTrace
<20	HCC	24	93.4% (89.5%-97.3%)
	Liver disease (LD)	36	HCC: 87.5% (79.6%-95.5%)<br>LD: 97.3% (95.5%-99.1%)
20-400	HCC	16	96.5% (94.1%-98.8%)
	Liver disease (LD)	12	HCC: 93.8% (89.6%-98.0%)<br>LD: 100.0% (100.0%-100.0%)
>400	HCC	21	100.00%
